# *Blastocystis* sp.: waterborne zoonotic organism, a possibility?

**DOI:** 10.1186/1756-3305-5-130

**Published:** 2012-06-28

**Authors:** Li Ii Lee, Tan Tian Chye, Biraj Man Karmacharya, Suresh Kumar Govind

**Affiliations:** 1 Department of Parasitology, Faculty of Medicine, University of Malaya, Kuala Lumpur, 50603, Malaysia; 2 Department of Community Programmes, Dhulikhel Hospital Kathmandu University Hospital, Dhulikhel Kavre, GPO Box 11008, Kathmandu, Nepal

**Keywords:** *Blastocystis*, Molecular evidence, Nepal, Rural communities, Waterborne zoonosis

## Abstract

**Background:**

*Blastocystis* sp. is a common intestinal parasite found in faecal sample surveys. Several studies have implicated human-to-human, zoonotic and waterborne transmissions by *Blastocystis* sp. However, there has been no study providing evidence interlinking these three transmissions in a community. We have previously shown a high prevalence of *Blastocystis* sp. subtype 4 amongst village dwellers in Bahunipati, Nepal, and the present study extends the observation to assess if the same subtype of *Blastocystis* sp. occurs in animals they rear and rivers they frequent.

**Methods:**

Faecal samples were collected from 65 animals. Four river water samples were collected from two rivers. Faecal samples were examined using *in vitro* cultivation. *Blastocystis* sp. from animal faecal and river samples were genotyped using seven subtype-specific sequence tagged site (STS) primer-polymerase chain reaction (PCR).

**Results:**

*Blastocystis* sp. infected 15.4% animals with subtype 4 being the predominant genotype (40.0%). Both rivers were contaminated with *Blastocystis* sp. subtype 1 and subtype 4, which were also detected in humans living in the same village in our previous study. *Blastocystis* sp. subtype 4 that was detected in buffalo and pigs was also found in the respective family members that reared these animals.

**Conclusions:**

This unusually high prevalence of *Blastocystis* subtype 4 found in village dwellers was also found to be pervasive in the animals they reared and the rivers they frequented implying a strong possibility of waterborne zoonosis for *Blastocystis* sp.

## Background

*Blastocystis* sp. is one of the most commonly found microorganisms infecting the intestine of humans and animals. Its occurrence in drinking water sources has been established through molecular analysis [1,2]. It exists as cyst, vacuolar, granular and amoebic forms and is believed to be transmitted through the faecal-oral route [3]. Several studies on the prevalence and genotyping of *Blastocystis* sp. in humans, animals or drinking water have been carried out throughout the world, from Asia, Australia, to Europe and America [3]. However, these reports were limited as they focused singly on either human-to-human, zoonotic or waterborne transmission. Thus, no comprehensive study has provided conclusive evidence interlinking these three transmissions in a community.

Human-to-human transmission by *Blastocystis hominis* subtype 3 has been reported in patients from two long-term health care facilities in Japan [4]. In Malaysia, we demonstrated a high prevalence of *Blastocystis* sp. in animal handlers [5] and more recently further substantial molecular evidence for zoonotic transmission was provided between animal and animal handlers in the Philippines and Australia [6,7]. In another study, *Blastocystis* sp. subtype 2 was detected in children and monkeys living within the same area in Kathmandu, Nepal [8]. Moreover, *Blastocystis* sp. was detected in sewage in Malaysia and Scotland [9] with two studies providing evidence for waterborne transmission by this parasite [1,2].

We have previously highlighted an unusually high prevalence of *Blastocystis* sp. subtype 4 in two villages, namely Bolde Phediche (76.9%; 30/39) and Bahunipati (95.8%; 23/24) in Nepal [10]. During the human faecal sample collection for the previous study in Bahunipati, we had also collected animal faecal sample and water samples. Therefore, we extended the observation in the present study by determining the prevalence of the organism in animal faeces and water sources in Bahunipati, where the prevalence of *Blastocystis* sp. of subtype 4 was higher in this rural community.

## Methods

### Study area and population

**Figure 1:**
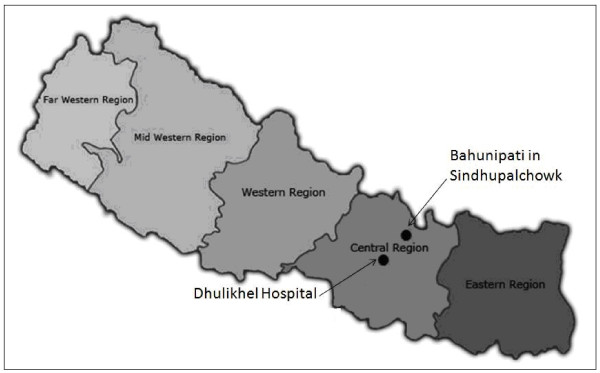
Study area in Bahunipati situated in Central Nepal.

A cross-sectional survey was conducted in a rural village located at Bahunipati in Sindhupalchowk District, Nepal, in November 2009 (Figure 1). This survey was carried out concurrently with human faecal sample collection in the previous study [10]. It is a hilly settlement with agricultural areas and animal farming activities being their livelihood. Faecal samples from 65 animals, including 19 buffaloes, six cows, 29 goats and 11 pigs were collected. These animals were reared by the respective villagers who participated in the previous study [10]. Ethical clearance for the present study was obtained from the Institutional Review Committee of Kathmandu University School of Medical Science/ Dhulikhel Hospital (IRC-KUSMS) with an ethical approval number of 04/10.

### Faecal examinations

Faecal examinations were performed by adding approximately 500 mg of faecal sample into Jones’ medium supplemented with 10% horse serum followed by incubation at 37°C and examined daily for the vacuolar form of *Blastocystis* sp. using light microscopy for the subsequent three days consecutively.

### Water sample concentration technique

A total of four water samples, one litre each, were collected from Indrawati River and Sindukhola River whereby two samples were collected from each river at two sampling points. The water sample collection was carried out concurrently with human faecal sample collection in the previous study [10]. Collected water samples were centrifuged at 1,400 × *g* for 10 minutes. Water concentrates were kept at −20°C until DNA extraction and further genotyping.

### Genotyping by polymerase chain reaction (PCR) using sequenced-tagged site (STS) primers

The preparation for genomic DNA extraction and genotyping using sequenced-tagged site (STS) primer with seven sets of specific STS primer for genotyping of *Blastocystis* from subtype 1 to subtype 7 [11] are as described in our previous study [10].

## Results and discussion

Our results showed that 10 animals (15.4%) were infected with *Blastocystis* sp. including four buffaloes, a cow, a goat and four pigs (Table 1a). *Blastocystis* sp. subtype 4 was the predominant subtype detected in animals (4/10; 40.0%; Table 2), which were found in a buffalo, a goat and two pigs. In contrast, the remaining six *Blastocystis* sp. isolates were not type-able with any STS primers used for this study. Therefore, they were categorised as unknown subtypes. All four water samples were contaminated with *Blastocystis* sp. (Table 1b) and subtype 1 (100.0%) was predominantly found followed by subtype 4 (3/4; 75%) (Table 2).

**Table 1:** **Prevalence of *Blastocystis* sp. infection amongst animals (a) reared by the villagers in Bahunipati, Nepal and prevalence of *Blastocystis* sp. contamination in the rivers (b)**

**(a)**	**Animals**	**No. of animal (%)**	**No. of animal infected by *Blastocystis* sp. (%)**
	Buffalo	19 (29.2)	4 (2.1)
	Cow	6 (9.2)	1 (1.7)
	Goat	29 (44.6)	1 (3.4)
	Pig	11 (16.9)	4 (36.4)
	Total	65 (100.0)	10 (15.4)
**(b)**	**River water**	**No. of sample**	**No. of sample contaminated with *Blastocystis sp.* **
	Sindukhola River	2 (50.0)	2 (50.0)
	Indrawati River	2 (50.0)	2 (50.0)
	Total	4 (100.0)	4 (100.0)

**Table 2:** **Subtype classification of *Blastocystis* sp. in Bahunipati obtained from animals and rivers**

	**Subtype classification**								
	**1**^**a**^	**2**	**3**	**4**^**a**^	**5**	**6**	**7**	**Unknown**	**1 + 4**^**b**^
Animals (10)	1	0	0	4	0	0	0	6	1
Waters (4)	4	0	0	3	0	0	0	0	3

^a^ Including mixed subtypes 1 + 4.

^b^*Blastocystis* sp. mixed subtypes infection.

The present study is timely, as recent The World Health Organization publications on drinking water quality have included *Blastocystis* sp. as one of the pathogens to be considered for waterborne zoonoses [12,13].

In the current study, it is interesting to note that every family from Bahunipati who participated in this survey owned animals, which were reared next to their dwelling. Prevalence of *Blastocystis* sp. infection in humans in Bahunipati (36.4%) is by far the highest [10] as compared to two other studies in Nepal, which reported 2.8% [14] and 25.6% [8] positive for *Blastocystis* sp.

*Blastocystis* sp. subtype 4 has been reported in guinea pig [15], lemur [16], opossum [17], rat [18,19] and woolly monkey [16]. However, our study found *Blastocystis* sp. subtype 4 in buffalo, goat and pig, which, previously have not been reported for these animals. Nonetheless, it has been reported that *Blastocystis* sp. subtype 1, 2, 3, 5, and 6 had been isolated from pig [20], but not subtype 4. From our observation, many of these studies were carried out on animals from the zoo and rarely involved livestock except some from Japan and the Philippines.

In this study, we observed that *Blastocystis* sp. subtype 4 that was detected in a buffalo and a pig was also found in its respective owner. These animals were reared at close proximity to their owners’ houses. In a study carried out in China, molecular-based evidence showed that *Blastocystis* sp. subtype 5 in pigs was also detectable in the humans who reared those pigs, suggesting that subtype 5 may be transmitted zoonotically [21]. Therefore, in the same light, our finding provides possible molecular-based evidence suggesting zoonotic potential of *Blastocystis* sp. subtype 4. It has also been reported in our previous study conducted in Malaysia that individuals who work closely with animals do stand at risk of acquiring *Blastocystis* sp. infection [5]. This finding was further supported by research done for faecal samples collected from animal facilities in the Philippines [6] and in Australia, Belgium and the Netherlands [7].

Our finding concurs with that by Leelayoova and colleagues [1], reporting the presence of *Blastocystis* sp. subtype 1 in drinking water. From our previous finding [10], it was shown that 37.5% of individuals in Bahunipati were infected by *Blastocystis* sp. subtype 1. The same subtype was shown to be present in two rivers that flowed beside the village in Bahunipati. Interestingly, both rivers were also contaminated with *Blastocystis* sp. subtype 4 while 95.8% of the communities in Bahunipati were infected by this subtype, which is the predominant subtype found in the community [10]. We observed that a poorly built and maintained septic tank was in close proximity to houses and the river, which could have facilitated seepage of *Blastocystis* sp. from faeces belonging to humans into the river and thus contaminating drinking water sources. Our previous study also revealed that both the older sister (15 years old) and younger brother (12 years old) in a same family were infected by *Blastocystis* sp. subtype 4, which could occur through various ways to facilitate human-to-human contamination.

**Figure 2:**
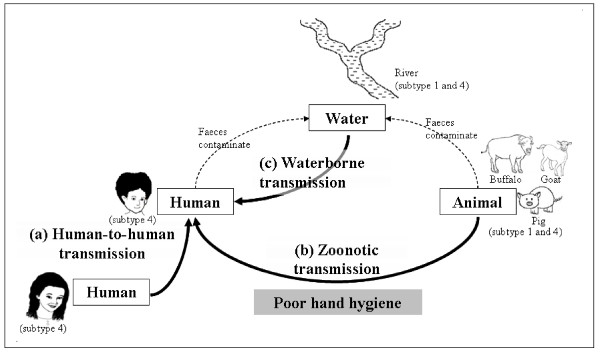
**Possible human-to-human, zoonotic and waterborne contaminations by *Blastocystis* sp. in Bahunipati, Nepal.**

Therefore, the molecular evidence presented in this study suggests that the Indrawati River and the Sindukhola River may be contaminated with faeces containing *Blastocystis* sp. subtype 4 from humans and *Blastocystis* sp. subtype 1 and 4 from animals. Since the contaminated rivers are the water sources for this community, drinking water from these rivers may facilitate waterborne contamination of *Blastocystis* sp. to them. In addition, poor hand hygiene could have facilitated the transmission from animals to humans. It is postulated that *Blastocystis* sp. subtype 4 that was pervasively found in humans, animals and rivers might be transmitted through human-to-human, zoonotic and waterborne contaminations. All three possible modes of contamination by *Blastocystis* sp. in Bahunipati, Nepal, are illustrated in Figure 2.

The current study is in agreement with that of Eroglu and Koltas [2], who reported the presence of *Blastocystis* sp. in infected patients, animals they reared and tap water, which was their source of drinking water. However, only *Blastocystis* sp. subtypes 1, 2 and 3 were detected in humans and animals, while only subtype 1 was detected in water in that study. *Blastocystis* sp. subtype 1 has also been reported in drinking water in a study conducted in Northern Thailand [1]. Therefore, the uniqueness of our current study is the detection of subtype 4 in animals and river water samples closely associated with village dwellers with an unusually high prevalence of subtype 4, not commonly seen in human populations. Even in studies where *Blastocystis* sp. subtype 4 was reported as the predominant subtype, it was found in patients [22,23] and not in rural communities [10].

## Conclusions

To the best of our knowledge, this is the first study to comprehensively provide molecular evidence supporting waterborne zoonotic transmission of *Blastocystis* sp. in a rural community. Specially, our data suggests the presence of waterborne and zoonotic transmissions of *Blastocystis* sp. subtype 4. We suggest that screening for *Blastocystis* sp. contamination needs to be performed as part of water quality assessments. Public education and proper management of livestock contamination may help reduce the incidence of *Blastocystis* sp. transmission in such communities.

## Competing interests

The authors declare that they have no competing interests.

## Authors’ contributions

LIL participated in the design of the study, performed all sample collection, examination of the samples, analysed the results and wrote the manuscript. TTC participated in the design of the study and revised the manuscript. BMK coordinated the research in Nepal. SKG conceived the study, participated in the design of the study and revised the manuscript. All authors read and approved the final version of the manuscript.
